# Macroautophagy Is Regulated by the UPR–Mediator CHOP and Accentuates the Phenotype of SBMA Mice

**DOI:** 10.1371/journal.pgen.1002321

**Published:** 2011-10-13

**Authors:** Zhigang Yu, Adrienne M. Wang, Hiroaki Adachi, Masahisa Katsuno, Gen Sobue, Zhenyu Yue, Diane M. Robins, Andrew P. Lieberman

**Affiliations:** 1 Department of Pathology, The University of Michigan Medical School, Ann Arbor, Michigan, United States of America; 2 Neuroscience Graduate Program, The University of Michigan Medical School, Ann Arbor, Michigan, United States of America; 3 Department of Neurology, Nagoya University, Nagoya, Japan; 4 Departments of Neurology and Neuroscience, Mount Sinai School of Medicine, New York, New York, United States of America; 5 Department of Human Genetics, The University of Michigan Medical School, Ann Arbor, Michigan, United States of America; Stanford University School of Medicine, United States of America

## Abstract

Altered protein homeostasis underlies degenerative diseases triggered by misfolded proteins, including spinal and bulbar muscular atrophy (SBMA), a neuromuscular disorder caused by a CAG/glutamine expansion in the androgen receptor. Here we show that the unfolded protein response (UPR), an ER protein quality control pathway, is induced in skeletal muscle from SBMA patients, AR113Q knock-in male mice, and surgically denervated wild-type mice. To probe the consequence of UPR induction, we deleted CHOP (C/EBP homologous protein), a transcription factor induced following ER stress. CHOP deficiency accentuated atrophy in both AR113Q and surgically denervated muscle through activation of macroautophagy, a lysosomal protein quality control pathway. Conversely, impaired autophagy due to Beclin-1 haploinsufficiency decreased muscle wasting and extended lifespan of AR113Q males, producing a significant and unexpected amelioration of the disease phenotype. Our findings highlight critical cross-talk between the UPR and macroautophagy, and they indicate that autophagy activation accentuates aspects of the SBMA phenotype.

## Author Summary

In many age-dependent neurodegenerative diseases, the accumulation of misfolded or mutant proteins drives pathogenesis. Several protein quality control pathways have emerged as central regulators of the turnover of these toxic proteins and therefore impact phenotypic severity. In spinal and bulbar muscular atrophy (SBMA), the mutant androgen receptor with an expanded glutamine tract undergoes hormone-dependent nuclear translocation, unfolding, and oligomerization—steps that are critical to the development of progressive proximal limb and bulbar muscle weakness in men. Here we show that the unfolded protein response (UPR), an endoplasmic reticulum stress response, is triggered in skeletal muscle from SBMA patients and knock-in mice. We find that disruption of the UPR exacerbates skeletal muscle atrophy through the induction of macroautophagy, a lysosomal protein quality pathway. In contrast, impaired autophagy diminishes muscle wasting and prolongs lifespan of SBMA mice. Our findings highlight cross-talk between the UPR and autophagy, and they suggest that limited activation of the autophagic pathway may be beneficial in certain neuromuscular diseases such as SBMA where the nucleus is the essential site of toxicity.

## Introduction

Many adult onset neurodegenerative disorders are characterized by the accumulation of abnormally folded proteins that self-associate into soluble oligomeric species or coalesce into insoluble protein aggregates. Among these disorders are ones caused by expansions of CAG/glutamine tracts [1], [2]. Spinal and bulbar muscular atrophy (SBMA), a member of this group, is a progressive neuromuscular disorder caused by an expanded glutamine tract near the amino terminus of the androgen receptor (AR) [3]. This mutation leads to hormone-dependent AR unfolding, and to the predominant loss of lower motor neurons in the brainstem and spinal cord of affected males. Clinical onset occurs in adolescence to adulthood and is characterized initially by muscle cramps and elevated serum creatine kinase [4], [5]. These myopathic features commonly precede muscle weakness, which inevitably develops as the disease progresses and is most severe in the proximal limb and bulbar muscles. Late in the course of disease, the pathologic features of SBMA include loss of motor neurons in the brainstem and spinal cord and the occurrence of myopathic and neurogenic changes in skeletal muscle 6,7.

Studies in mouse models have defined several general principles that guide our understanding of SBMA pathogenesis. Transgenic over-expression of the expanded glutamine AR leads to disease, consistent with the notion that toxicity is predominantly mediated through a gain-of-function mechanism [8], [9]. This toxicity is androgen-dependent in mice and in SBMA patients, an observation that led to recent clinical trials with anti-androgens [10]–[12]. To model SBMA in mice, our laboratory used gene targeting to exchange 1340 bp of mouse *Ar* exon 1 with human sequence containing 21 or 113 CAG repeats [13], [14]. Mice expressing the expanded glutamine AR (AR113Q) develop androgen-dependent neuromuscular and systemic pathology that models SBMA [14], [15], whereas AR21Q males are similar to wild type littermates [13], [14]. In AR113Q mice, denervation and muscle pathology occur early in life, prior to detectable motor neuron loss, indicating that neuronal dysfunction or distal axonal degeneration and myopathy are early disease manifestations. The notion that pathology arising in muscle contributes to disease is consistent with findings in transgenic mice in which over-expression of the wild type AR in skeletal muscle leads to hormone-dependent myopathy and motor axon loss [16], and with data showing a rescue of the disease phenotype in SBMA transgenic mice by over-expressing IGF-1 in skeletal muscle [17]. Taken together, these observations focused our attention on the role of skeletal muscle in disease pathogenesis.

The cellular pathways by which the expanded glutamine AR mediates toxicity are complex and incompletely understood, with evidence in several model systems showing disruption of gene expression [18]–[23], alterations in RNA splicing [24], impairments in axonal transport [25]–[27] and defects in mitochondrial function [28]. Toxicity likely results from the cumulative effects of altering a diverse array of cellular processes, indicating that potential treatments targeting a single downstream pathway are likely to be unsuccessful. These findings prompted us to concentrate instead on understanding the proximal mechanisms that regulate degradation of the mutant protein. Work in cellular and mouse models has established that degradation and aggregation of the polyglutamine AR are regulated by the Hsp90-based chaperone machinery [29], [30], and that manipulating the expression or function of Hsp70-dependent E3 ubiquitin ligases markedly affects AR turnover through the ubiquitin-proteasome pathway [31]–[33].

In addition to the chaperone machinery, other pathways regulating protein quality control have been implicated in SBMA pathogenesis. Here we explored the role of the unfolded protein response (UPR), an integrated signal transduction pathway that transmits information about protein folding within the ER lumen to the nucleus and cytosol to regulate protein synthesis and folding and to influence cell survival [34], [35]. Prior studies showed that amino-terminal fragments of the polyglutamine AR activate the UPR *in vitro* [36], but little is known about the role of this pathway in more complex models of disease. We now show that the UPR is activated in skeletal muscle from SBMA patients and AR113Q mice. Moreover, genetic disruption of the ER stress response by deletion of the gene encoding the transcription factor C/EBP homologous protein (CHOP), a mediator of the UPR [34], accentuates skeletal muscle atrophy in AR113Q mice. Further, we show that enhanced muscle wasting in the setting of CHOP deficiency is due to increased macroautophagy (hereafter referred to as autophagy), a lysosomal protein quality control pathway implicated in the pathogenesis of polyglutamine and motor neuron diseases. In contrast, diminished autophagy due to Beclin-1 haploinsufficiency decreased muscle wasting and extended the lifespan of AR113Q males, unexpectedly ameliorating the disease phenotype. Our findings highlight cross-talk between the UPR and autophagy, and demonstrate that increased autophagy promotes atrophy of SBMA muscle.

## Results

### The UPR is activated in SBMA muscle

**Figure 1:**
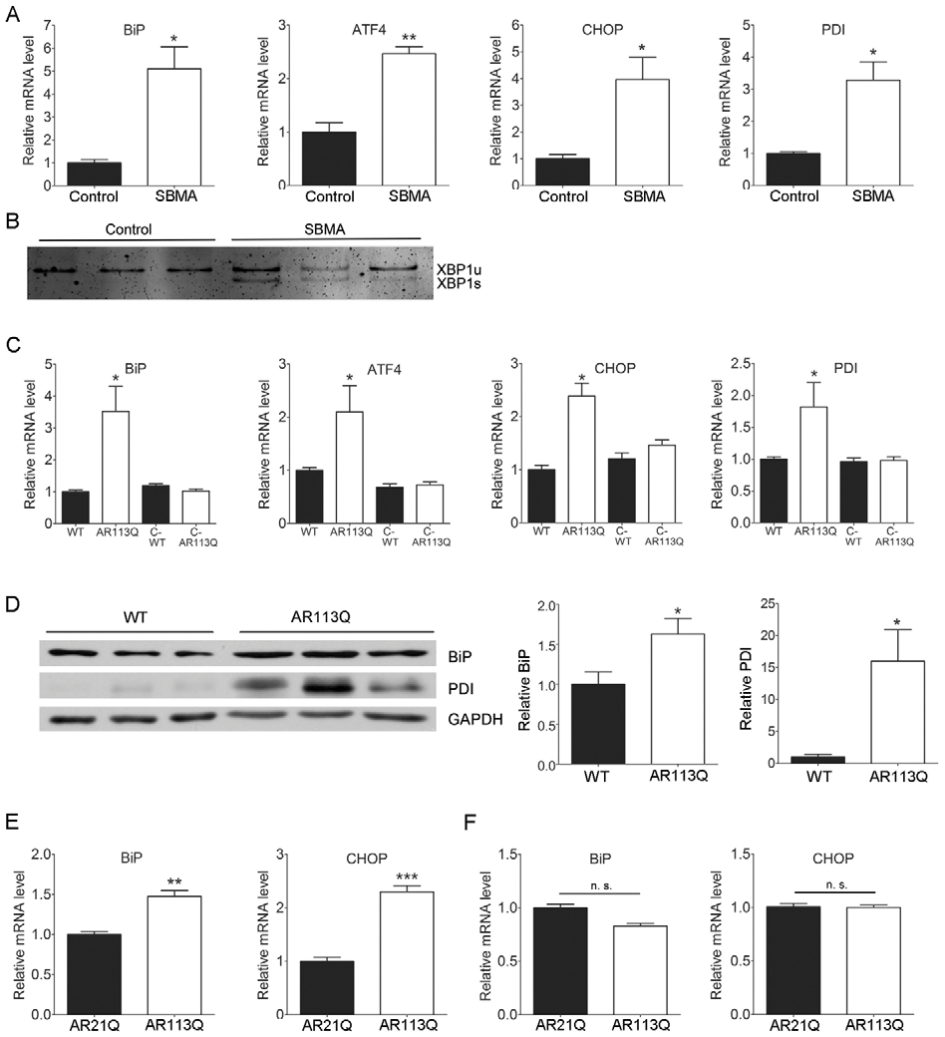
The UPR is activated in SBMA muscle. A. Relative mRNA expression in skeletal muscle from SBMA patients (white bars, *n* = 3) and controls (black bars, *n* = 3) (mean +/− SEM). * p<0.05, ** p<0.01 by Student's *t* test. B. Splicing of XBP1 mRNA was assessed by RT-PCR. Products from unspliced (XBP1u) and spliced (XBP1s) transcripts were resolved on a nondenaturing polyacrylamide gel and stained with SYBR green. C. Relative mRNA expression in proximal hind limb muscle (mean +/− SEM). Mice evaluated were littermate WT (*n* = 6), AR113Q (*n* = 6), castrated WT (C-WT, *n* = 6) and castrated AR113Q males (C-AR113Q, *n* = 5) on a mixed C57BL/6J-129 genetic background. *p<0.05 by ANOVA. D. Western blot of BiP and PDI expression in proximal hind limb muscle. Right panels show quantification of signal relative to loading control (mean +/− SEM). * p<0.05 by Student's *t* test. E. Relative mRNA expression in proximal hind limb muscle of AR21Q (*n* = 5) and AR113Q (*n* = 3) males backcrossed to C57BL/6J. ** p<0.01, ***p<0.001 by Student's *t* test. F. Relative mRNA expression in spinal cord of AR21Q (*n* = 5) and AR113Q (*n* = 3) males (mean +/− SEM). n. s. = not significant by Student's *t* test.

To determine whether the ER stress response is activated in SBMA we obtained skeletal muscle from patients and male controls. Gene expression analysis by qPCR demonstrated that SBMA muscle contained significantly higher levels of several mRNAs that are induced in response to ER stress (Figure 1A) [34], [35]. These encoded the ER chaperone immunoglobulin binding protein (BiP), the transcription factors activating transcription factor-4 (ATF4) and its target CHOP, and the ER folding enzyme protein disulfide isomerase (PDI). Further, increased splicing of mRNA encoding X-box binding protein-1 (XBP1) was detected (Figure 1B), indicating that activation of the proximal UPR sensor inositol-requiring protein-1 (IRE1) had occurred.

Analysis of proximal hind limb muscle from adult AR113Q male mice similarly demonstrated the induction of mRNAs encoding BiP, ATF4, CHOP and PDI (Figure 1C). This was associated with increased expression of BiP and PDI proteins, as demonstrated by western blot (Figure 1D). As the neuromuscular phenotype of these mice is both hormone and glutamine-length dependent [14], we sought to determine whether the occurrence of ER stress was similarly regulated. Surgical castration at 5–6 wks ameliorated the induction of these transcripts in adult AR113Q males, demonstrating that UPR activation was responsive to levels of circulating androgens (Figure 1C). Further, direct comparison with mice generated using the same gene targeting strategy but with only 21 CAG repeats in the *Ar* gene [13] confirmed that UPR activation was dependent upon the presence of an expanded glutamine tract (Figure 1E). In contrast, we did not detect induction of ER stress-induced mRNAs such as BiP and CHOP in spinal cords of AR113Q males (Figure 1F), nor did we detect increased expression of BiP or PDI proteins in spinal motor neurons (not shown). We conclude that the UPR is activated in skeletal muscle from SBMA patients and knock-in mice.

### CHOP deletion increases AR113Q muscle atrophy and activates autophagy

As the UPR plays a central role in protein homeostasis in the ER and influences survival in a cellular model of SBMA [36], we sought to determine its role in disease pathogenesis *in vivo*. To accomplish this, we generated AR113Q males deficient in CHOP, a regulator of cell survival during ER stress that we found to be up-regulated in SBMA muscle. CHOP null mice exhibit impaired programmed cell death following pharmacological induction of ER stress [37]. Further, CHOP deficiency accentuates the phenotype of Pelizaeus-Merzbacher Disease mice [38] yet rescues the motor deficits of Charcot-Marie-Tooth 1B mice [39], demonstrating that deletion of this transcription factor is an informative approach to probing the role of the UPR in model systems. Notably, CHOP null mice do not display neuromuscular pathology, thereby enabling us to assess the outcome of genetic disruption of the UPR on the SBMA phenotype.

**Figure 2:**
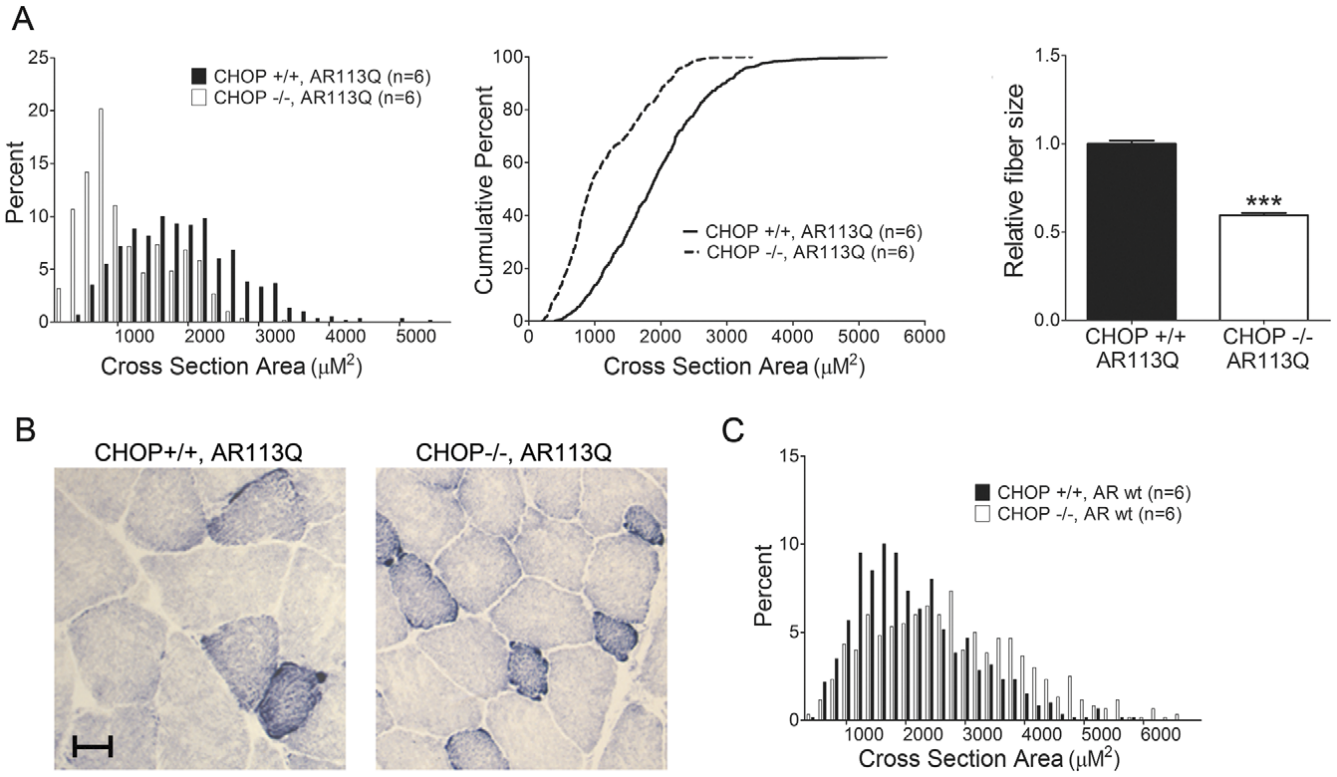
CHOP deletion accentuates muscle atrophy in AR113Q mice. A. Muscle fiber size (100 fibers/mouse) was quantified from proximal hind limb muscle of AR113Q (black) or AR113Q, CHOP −/− mice (white) at 12 wks. Left panel shows fiber size distribution, middle panel shows cumulative percent of fibers as a function of fiber area, and right panel shows relative fiber cross sectional area (mean +/− SEM). Left, middle panels, p<0.0001 by Mann-Whitney test. Right panel, p<0.001 by Student's *t* test. B. Representative image of muscle fibers following NADH stain. Bar = 20 µM. C. Distribution of proximal hind limb muscle fiber size from wt (black) and CHOP −/− (white) mice at 12 wks. Difference not significant by Mann-Whitney test.

CHOP deficiency markedly affected AR113Q muscle, the site of UPR activation, by accentuating skeletal muscle atrophy (Figure 2A, 2B). This unexpected effect on muscle fiber size yielded a significant shift in the distribution of fibers towards a smaller cross sectional area, resulting in a mean fiber size that was ∼1/3 smaller than that measured in AR113Q males. In contrast, CHOP null males expressing the wild type AR had muscle fibers that were similarly sized to age matched wild type males (Figure 2C). Although CHOP deficiency did not alter AR113Q total body mass or survival (not shown), our data show that disruption of the UPR by CHOP deletion increased muscle wasting in AR113Q male mice.

**Figure 3:**
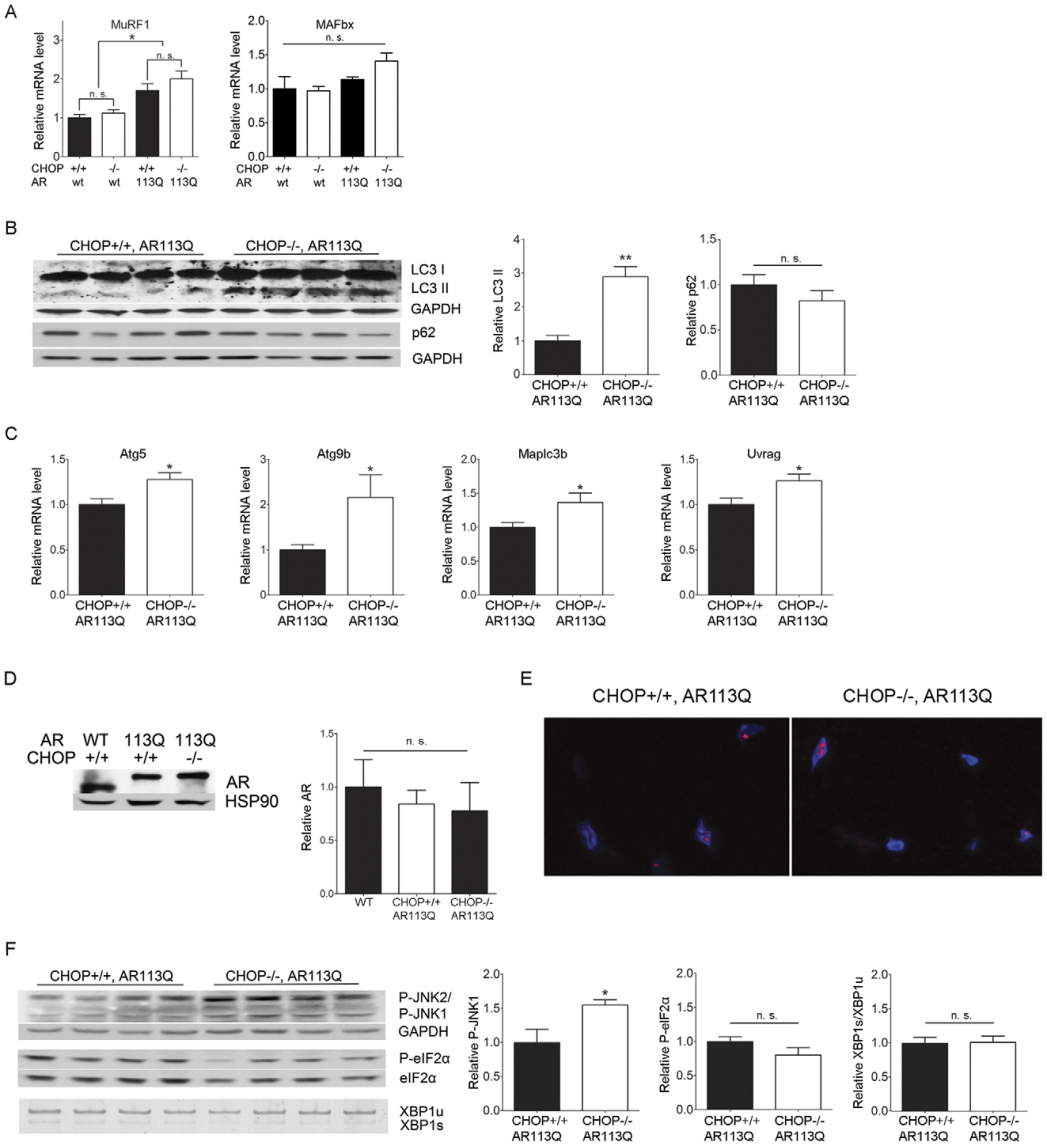
Autophagy is increased in AR113Q, CHOP −/− muscle. A. Relative expression of MurRF1 and MAFbx mRNAs in proximal hind limb muscle of 12 wk mice (*n* = 5–6/genotype). *p<0.05 by ANOVA, n. s. = not significant. B. LC3 and p62 expression in proximal hind limb muscle of 12 wk mice was assessed by western blot. Right panels show quantification of signal relative to GAPDH. **p<0.01 by Student's *t* test. C. Relative expression of mRNAs encoding autophagy regulators in proximal hind limb muscle. *p<0.05 by Student's *t* test. C. Androgen receptor protein expression in skeletal muscle of 12 wk mice. Hsp90 serves as a loading control. Right panel shows quantification of relative signal intensity (*n* = 3/genotype). D. Proximal hind limb muscles stained for the androgen receptor (in red) exhibit intranuclear inclusions. Nuclei are stained by DAPI (in blue). E. P-JNK and P-eIF2 alpha expression (top, middle) and XBP1 mRNA splicing (bottom) in proximal hind limb muscle of 12 wk mice. Right panels show quantification of signal relative to loading control. **p<0.01 by Student's *t* test. n. s. = not significant.

To determine the mechanism by which CHOP deficiency increased skeletal muscle atrophy, we initially considered the possibility that motor neuron degeneration was more severe in AR113Q mice deficient in CHOP, resulting in enhanced neurogenic atrophy. However, we found no evidence of increased motor neuron loss in the spinal cords of these double mutants (not shown). Furthermore, skeletal muscle expression of mRNAs induced following denervation [40], including those encoding myogenin and MyoD, was similar in AR113Q and AR113Q, CHOP null males (Figure S1). These findings suggested that enhanced muscle atrophy in animals deficient in CHOP was not mediated by increased motor neuron loss, but rather reflected augmented activation of a pathway that mediates muscle wasting. To directly test this notion, we first examined the expression of muscle RING-finger protein 1 (MuRF1) and Atrogin1/Muscle Atrophy F-box (MAFbx) (Figure 3A), two E3 ubiquitin ligases that are induced in atrophying skeletal muscle and mediate enhanced protein degradation through the proteasome [41]. While modest induction of MuRF1 mRNA was observed in AR113Q muscle, its expression was not further increased by CHOP deficiency. No significant change in MAFbx expression was detected. Additionally, CHOP deficiency did not alter expression of the 20S proteasome subunit in skeletal muscle (Figure S2). We conclude that enhanced atrophy of hind limb muscle in AR113Q, CHOP null mice was not associated with a significant induction of E3 ligases that promote muscle protein degradation through the ubiquitin-proteasome pathway.

These findings prompted us to consider the possibility that another protein degradation pathway underlies the increased atrophy triggered by CHOP deficiency. As recent studies demonstrate that autophagy contributes to skeletal muscle wasting [42], we next examined the activity of the autophagic pathway following disruption of the UPR. Western blot demonstrated a ∼3-fold increase in the autophagosome marker LC3-II (microtubule-associated protein 1, light chain 3-II) in skeletal muscle from AR113Q, CHOP null mice (Figure 3B). No accumulation of p62 was detected (Figure 3B) consistent with the notion that flux through the autophagic pathway was intact following disruption of the UPR. Consistent with the notion that CHOP deficiency induced autophagy in AR113Q muscle, we detected increased expression of mRNAs encoding the autophagy regulators Atg5, Atg9B, LC3B and UVRAG (Figure 3C). Notably, induction of autophagy was not associated with altered levels of AR protein (Figure 3D) or the appearance of AR immunoreactive intranuclear inclusions in skeletal muscle nuclei (Figure 3E). These observations are consistent with a prior report demonstrating that the androgen receptor largely escapes autophagic degradation following its translocation into the nucleus [43], and indicate that enhanced muscle atrophy in CHOP null mice is independent of changes in AR protein levels. CHOP deficiency did not alter phosphorylation of eukaryotic translation initiation factor 2 alpha (eIF2 alpha) or splicing of XBP1 mRNA (Figure 3F), signals generated by the proximal UPR sensors protein kinase RNA-like ER kinase (PERK) and IRE1 that have been linked to the regulation of autophagy [44], [45]. In contrast, we observed a modest, but significant increase in the phosphorylation of c-Jun N-terminal kinases (JNK) (Figure 3F), suggesting that signaling through JNK may contribute to enhanced activation of autophagy in AR113Q, CHOP null muscle, as observed in other systems [46].

### CHOP deficiency increases autophagy-induced atrophy of denervated muscle

**Figure 4:**
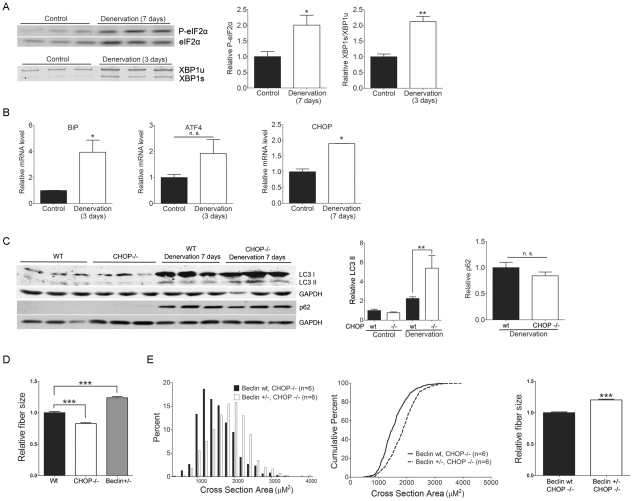
CHOP deficiency increases denervation-induced atrophy through autophagy. Denervated gastrocnemius muscles or contralateral intact controls were harvested at the indicated times following unilateral sciatic nerve transection in 6 wk male mice. A. Western blot shows enhanced eIF2 alpha phosphorylation (top) and RT-PCR demonstrates increased XBP1 mRNA splicing (bottom) in denervated muscle. Right panels show relative quantification of signal intensity. *p<0.05 by Student's *t* test. B. Relative expression of BiP, ATF4 and CHOP mRNA (*n* = 3). *p<0.05 by Student's *t* test. C. Following surgical denervation of wild type or CHOP−/− mice, LC3 and p62 expression was assessed by western blot. Right panels show quantification of signal relative to GAPDH. ***p<0.001 by ANOVA, n. s. = not significant. D. Muscle fiber size (100 fibers/mouse) was quantified from wild type (black, *n* = 5), CHOP −/− (white, *n* = 3) and Beclin-1 +/− (grey, *n* = 3) mice 7 days post sciatic nerve transection. Shown is relative fiber cross sectional area (mean +/− SEM). ***p<0.001 by ANOVA. E. Muscle fiber size (100 fibers/mouse) was quantified from CHOP −/− (black, *n* = 6) or CHOP −/−, Beclin-1 +/− mice (white, *n* = 6) 7 days post sciatic nerve transection. Left panel shows fiber size distribution, middle panel shows cumulative percent of fibers as a function of fiber area, and right panel shows relative fiber cross sectional area (mean +/− SEM). Left, middle panels, p<0.0001 by Mann-Whitney test. Right panel, p<0.001 by Student's *t* test.

Our observation of robust UPR activation in AR113Q skeletal muscle raised the possibility that muscle denervation induces ER stress, and that disruption of the UPR by CHOP deficiency enhances wasting by altering the cellular response to ER stress. To first test whether denervation is sufficient to activate the UPR in skeletal muscle, wild type male mice underwent unilateral sciatic nerve transection, and denervated and intact gastrocnemius muscles were harvested at 3 or 7 days post surgery. Denervation significantly increased phosphorylation of eIF2 alpha and splicing of XBP1 mRNA (Figure 4A) indicating that activation of the proximal UPR sensors PERK and IRE1 had occurred. Further, gene expression analysis by qPCR demonstrated a significant induction of BiP and CHOP mRNAs in denervated muscle, while ATF4 mRNA levels exhibited a similar trend that failed to reach statistical significance (Figure 4B). We conclude that denervation activated the UPR in skeletal muscle.

These results encouraged us to use this system to further explore the relationship between the UPR and autophagy, and to test the notion that CHOP deficiency enhances muscle wasting through the induction of autophagy. Surgical denervation of male mice expressing the wild type AR demonstrated that CHOP deficiency significantly increased activity of the autophagic pathway, similar to our findings in AR113Q muscle. Denervated CHOP null muscle harvested 7 days post surgery contained ∼2.5 fold more LC3-II than did wild type muscle (Figure 4C). p62 did not accumulate in CHOP deficient muscle, indicating that flux through the autophagic pathway was intact. CHOP deficiency also accentuated skeletal muscle atrophy following denervation, producing a significant decrease in mean fiber size (Figure 4D). Our findings demonstrate that CHOP deficiency enhances autophagy and increases muscle wasting following denervation.

To confirm that autophagy contributes to muscle atrophy following surgical denervation, we transected the sciatic nerve of Beclin-1 haploinsufficient male mice [47]. Beclin-1 (encoded by *Becn1*) is a critical regulator of autophagy that binds class III phosphoinositide 3-kinase and is both required for the initiation of autophagosome formation and contributes to autophagosome maturation [48]. Mice haploinsufficient for Beclin-1 form fewer autophagosomes in skeletal muscle [49] and therefore allowed us to probe the role of autophagy in the response of muscle to sciatic nerve transection. Muscle haploinsufficient for Beclin-1 exhibited significantly increased mean fiber size compared to either wild type or CHOP null muscle following surgical denervation (Figure 4D) supporting a role for autophagy in muscle wasting. To directly test the notion that CHOP deficiency enhanced muscle wasting by activating autophagy, we generated CHOP null mice haploinsufficient for Beclin-1 (Figure 4E). Following denervation, these mice exhibited significantly less atrophy than CHOP null males, demonstrating that the effects of CHOP deficiency on muscle wasting were mediated through autophagy.

### Beclin-1 haploinsufficiency attenuates the phenotype of AR113Q males

**Figure 5:**
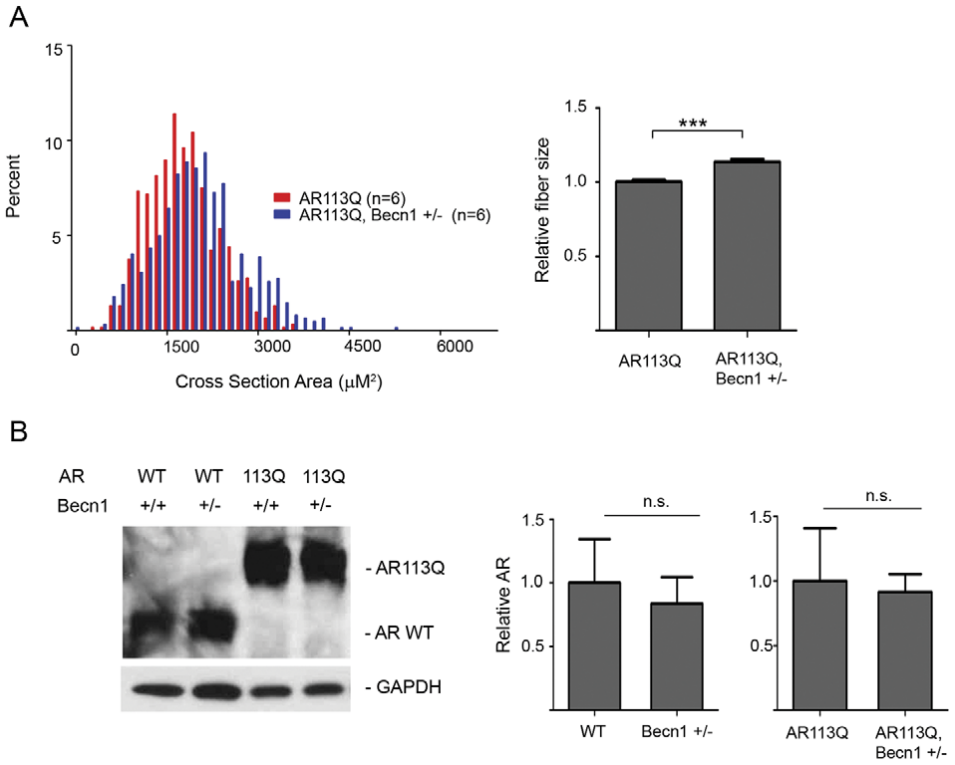
Effects of Beclin-1 haploinsufficiency on AR113Q muscle. A. Muscle fiber size (100 fibers/mouse) was quantified from proximal hind limb muscle of AR113Q (red, *n* = 6) or AR113Q, Beclin-1 +/− (blue, *n* = 6) mice at 16 wks. Left panel shows fiber size distribution, and right panel shows relative fiber cross sectional area (mean +/− SEM). Left panel, p<0.0001 by Mann-Whitney test. Right panel, p<0.0001 by Student's *t* test. B. AR expression in skeletal muscle of 16 wk mice was assessed by western blot. GAPDH controls for loading. Right panel shows quantification of signal relative to GAPDH (mean +/− SEM). Differences not significant (n. s.).

Our finding that enhanced autophagy triggered by CHOP deficiency promoted muscle wasting in AR113Q mice prompted us to determine the consequences of limiting autophagy on the SBMA phenotype. To accomplish this, we generated AR113Q males haploinsufficient for Beclin-1. Similar to effects following surgical denervation, Beclin-1 haploinsufficiency significantly increased AR113Q muscle fiber size, although in this case the effect was less robust (Figure 5A). Limiting activity of the autophagic pathway did not alter levels of either the wild type or polyglutamine AR protein (Figure 5B), consistent with the notion that other protein quality control pathways, such as the proteasome, degrade the receptor once localized to the nucleus.

**Figure 6:**
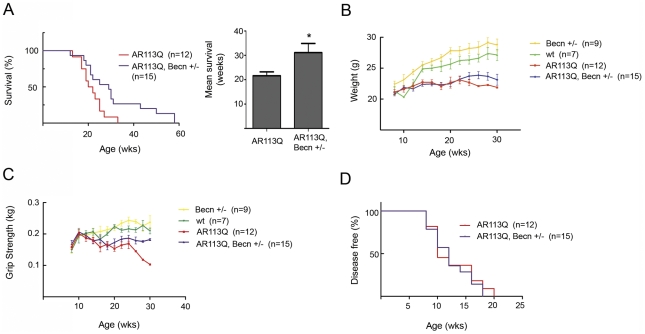
Beclin-1 haploinsufficiency extends lifespan in AR113Q males. A. Left panel, Kaplan-Meyer survival curve of AR113Q males (red line, *n* = 12) and AR113Q, Beclin-1 +/− males (blue line, *n* = 15). *p<0.05 by log-rank analysis. Right panel, mean survival +/− SEM. *p<0.05 by Student's *t*-test. B, C. Body weight (panel B) and grip strength (panel C) at different ages for wild type (wt, green line, *n* = 7), Beclin-1 +/− (yellow line *n* = 9), AR113Q (red line, *n* = 12), and AR113Q, Beclin-1 +/− (blue line, *n* = 15) male mice. D. Age of disease onset as measured by a decrease of 5% or more in forelimb grip strength (not significant by log rank analysis).

Despite the limited changes in AR113Q muscle, Beclin-1 haploinsufficiency had a striking effect on survival. The lifespan of AR113Q males haploinsufficient for Beclin-1 was extended on average by ∼10 wks compared to AR113Q, Beclin-1 wild type littermates (Figure 6A). AR113Q males exhibited a mean survival of 21.6 wks; Beclin-1 haploinsufficiency extended mean lifespan by ∼44% to 31.1 wks. Lifespan extension was not associated with rescue to wild type levels of body mass or motor performance as measured by grip strength (Figure 6B, 6C). However, AR113Q males haploinsufficient for Beclin-1 aged over 20 weeks maintained motor function while AR113Q, Beclin-1 wild type littermates exhibited a marked drop-off (Figure 6C). Consistent with the notion that the effects of Beclin-1 haploinsufficiency on motor function were most manifest in older mice, we found no change in the age of disease onset (defined as the point at which grip strength was 5% less than controls) due to Beclin-1 haploinsufficiency (Figure 6D). Our data indicate that Beclin-1 haploinsufficiency significantly extended the duration of disease by prolonging survival and maintaining motor function of SBMA mice.

## Discussion

The accumulation of misfolded, mutant proteins is a common basis for adult onset neurodegenerative diseases including those caused by CAG/glutamine tract expansions [1], [2], and pathways controlling protein homeostasis are central to the cellular response to these stressors. Here we investigated the role of the UPR, a regulator of ER protein quality control [34], [35], in the pathogenesis of SBMA, a neuromuscular disease caused by a glutamine tract expansion in the AR. Our findings demonstrate the occurrence of ER stress in skeletal muscle from SBMA patients, AR113Q mice and wild type mice following surgical denervation. To identify the functional consequence of this response, we generated AR113Q mice deficient in the UPR-mediator CHOP, a transcription factor induced downstream of ATF4 following ER stress. We show that CHOP deletion accentuates muscle atrophy in both AR113Q mice and in surgically denervated wild type males. Notably, in both cases, enhanced muscle wasting due to CHOP deficiency is mediated by increased autophagy, a lysosomal protein quality control pathway that has emerged as a central regulator of proteostasis in several protein aggregation neurodegenerative diseases. While CHOP deficiency activates autophagy and enhances muscle wasting in SBMA mice, limiting autophagy by Beclin-1 haploinsufficiency diminishes muscle atrophy, maintains motor function in aged animals and markedly extends lifespan. Our data highlight the central role of the UPR in remodeling the activity of the protein quality control machinery, and indicate that robust activation of autophagy accentuates the muscle atrophy of SBMA.

Activation of the UPR has been reported previously in yeast and mammalian cell culture models of polyglutamine disease [36], [50], [51], and the induction of ER stress responsive transcripts has been noted in Huntington disease mice [52]. The findings reported here extend these observations, demonstrating that the ER stress response is triggered in skeletal muscle from both SBMA patients and knock-in mice. Further, we define new aspects of the functional link between the UPR and autophagy. Several mechanisms by which the UPR regulates autophagy have been proposed based on studies in mammalian models, but a role for CHOP has not been identified previously. Data from a cellular model of polyglutamine disease indicate that phosphorylation of eIF2 alpha by PERK mediates the induction of LC3-II [45], while a recent study in cellular and mouse models of superoxide dismutase 1 (SOD1)-linked ALS show that XBP1 deletion activates autophagy [44]. As CHOP deficiency altered neither phosphorylation of eIF2 alpha nor splicing of XBP1 in AR113Q mice, we suggest that the effects identified here occur through a distinct mechanism. JNK, a downstream target of IRE1 [53], can also stimulate LC3-II formation [46], and the occurrence of increased JNK phosphorylation in AR113Q, CHOP null muscle raises the possibility that this signaling pathway contributes to autophagy activation.

The functional consequences of altered autophagy in SBMA mice were unexpected and suggest that limiting activity of this pathway is beneficial for certain aspects of the disease phenotype. As the polyglutamine AR resides in the nucleus in the presence of ligand and largely escapes degradation through this pathway [43], we found that soluble and aggregated species of the mutant AR do not change when mice are deficient in CHOP or haploinsufficient for Beclin-1. We suggest that this reflects predominant degradation of the AR by the proteasome, a protein quality control pathway active in the nucleus. The extension of AR113Q lifespan by Beclin-1 haploinsufficiency contrasts with findings in Drosophila showing that disruption of autophagy exacerbates degeneration when the polyglutamine AR is expressed in the eye [54]. This difference may reflect variations in the extent to which autophagy is disrupted, as Beclin-1 haploinsufficiency decreases autophagosome number but does not completely block this pathway. Additionally, small molecule activators of autophagy reportedly promote survival of cultured motor neurons expressing the polyglutamine AR [43], raising the possibility that the findings described here in AR113Q mice reflect predominant effects outside the CNS, such as in skeletal muscle. While activation of autophagy following UPR disruption exacerbates atrophy of SBMA muscle in mice, recent studies in SOD1 models of ALS show that autophagy induction following XBP1 deletion ameliorates the disease phenotype [44]. Mutant SOD1, a cytosolic protein, is a target for autophagic degradation and stimulating this pathway clears aggregates of the mutant protein.

Of the clinical symptoms experienced by SBMA patients, muscle wasting is a substantial contributor to morbidity. Here we show that activation of autophagy significantly enhances atrophy of surgically denervated and AR113Q muscle. In contrast, limiting autophagy prolongs lifespan and maintains motor function in SBMA mice. While the effects of Beclin-1 haploinsufficiency are relatively mild in AR113Q muscle, lifespan extension is striking, and likely reflects benefits of limited autophagy in cell types other than muscle fibers, perhaps including effects on metabolism. Defining the targets affected by Beclin-1 haploinsufficiency that mediate lifespan extension remains an important goal for future work. Notably, strategies to modulate the activity of the autophagic pathway have attracted considerable attention as studies in several polyglutamine disease models have documented degradation of cytoplasmic protein aggregates through autophagy [55]. Efforts are now underway to identify small molecules that activate the autophagic pathway in hopes of ameliorating the phenotypes of these diseases [56], [57]. Our data suggest that autophagy activators are unlikely to be effective therapeutics for the subset of protein aggregation disorders where nuclear localization of the mutant protein is required for toxicity. Furthermore, in SBMA, the effects of disease on muscle may be accentuated by activation of autophagy. We suggest that alternative approaches to stimulate other components of the protein quality control machinery, such as the Hsp90-based chaperone machinery, are more likely to yield clinical benefits in SBMA and related protein aggregation disorders.

## Materials and Methods

### Mice

Derivation of mice with targeted *Ar* alleles containing 21 or 113 CAG repeats in exon 1 was described previously [14], [15]. Briefly, mice were generated by recombining a portion of human exon 1 encompassing amino acids 31–484 with the mouse *Ar* gene in CJ7 embryonic stem cells. Male chimeras were mated with C57BL/6J females, and females heterozygous for the targeted *Ar* allele were backcrossed to C57BL/6J to generate mice used in this study. Surgical castration of 5–6 wk old males was as previously described [14]. Unless otherwise specified, skeletal muscles were harvested from adult AR113Q male mice at 3–5 months, except from castrated AR113Q males, in which case animals were 18 months of age. CHOP deficient mice (B6.129S-Ddit3^tm1Dron^/J) [37] were purchased from the Jackson Laboratory and backcrossed to C57BL/6J ten or more generations. Mice with a *Becn1* null allele were previously reported [47] and backcrossed to C57BL/6J ten or more generations. All procedures involving mice were approved by the University of Michigan Committee on Use and Care of Animals, in accord with the NIH Guidelines for the Care and Use of Experimental Animals.

### Sciatic nerve transection

7 wk old C57BL/6J, CHOP deficient or *Becn1* haploinsufficient male mice congenic to C57BL6/J were used for studies of denervated muscle. Under deep inhaled anesthesia with 2% isoflurane, the right sciatic nerve was exposed at the thigh just below the sciatic notch. Both the proximal and distal sides were ligated with monocryl 4-0 suture, and about 2 mm of sciatic nerve was cut between the ligations to prevent axonal regeneration. At 3 and 7 days after surgery, the right gastrocnemius and tibialis anterior muscle were dissected and frozen for histology or RNA and protein analysis. The contralateral side was used as control.

### Human skeletal muscle samples

Anonymized SBMA muscle and control biopsy samples were obtained from the University of Michigan Medical School in accordance with IRB procedures and in a manner that assured patient privacy. Additionally, anonymized skeletal muscle was harvested from SBMA patients at autopsy, as approved by the ethics committee of the Nagoya University Graduate School of Medicine and in accordance with the Declaration of Helsinki (Hong Kong Amendment).

### Muscle fiber size quantification

Muscle was frozen in isopentane chilled by liquid nitrogen, cut in cross section at a thickness of 5 µm and stained by H&E. Digital images were captured using a Zeiss Axioplan 2 imaging system. The area of each muscle fiber was defined using Adobe Photoshop CS4 or ImageJ, and the pixel number was converted to µm^2^ according to scale. 100 adjacent fibers from each section were measured.

### RNA analysis

Total RNA isolated from tissues with Trizol (Invitrogen, Carlsbad, CA) served as a template for cDNA synthesis using the high capacity cDNA archive kit from Applied Biosystems (Foster City, CA). Gene-specific primers and FAM labeled probes (Human: BiP, Hs99999174_m1; CHOP, Hs99999172_m1; ATF4, Hs00909568_g1; PDI, Hs00168586_m1; Mouse: BiP, Mm00517691_m1; CHOP, Mm00492097_m1; ATF4, Mm00515324_m1; PDI: Mm01243184_m1; MAFbx, Mm00499518_m1; MuRF1, Mm01185221_m1; α-acetylcholine receptor, Mm00431627_m1; Myod1, Mm00440387_m1; Myog, Mm00446194_m1; Atg5, Mm00504340_m1; Atg9b, Mm01157883_g1; Maplc3b, Mm00782868_sH; Uvrag, Mm00724370_m1) were purchased from Applied Biosystems. TaqMan assays were performed in duplicate using 5 ng aliquots of cDNA on an ABI 7500 Real Time PCR system. Relative expression levels were calculated comparing with the expression of 18S rRNA. Semi-quantitative RT-PCR analysis of Xbp1 RNA splicing was performed using primers (mouse: 5′-GAACCAGGAGTTAAGAAC-3′ and 5′-AGGCAACAGTGTCAGAGT-3′; human: 5′-GAATGAGTGAGCTGGAACAG-3′ and 5′-GAGTCAATACCGCCAGAATC-3′) to amplify 10 ng of cDNA through 22 cycles. One tenth of the total PCR products were resolved on 15% nondenaturing polyacrylamide gels and stained with SYBR Green 1 (Invitrogen, Eugene, OR) after electrophoresis. Bands were visualized on a Typhoon Trio+ scanner (Amersham Biosciences, Pistcataway, NJ) and analyzed with AlphaImager 2200 software (Alpha Innotech Corporation, San Leandro, CA).

### Protein expression analysis

Muscle tissue was homogenized in RIPA buffer containing complete protease inhibitor cocktail (Roche, Indianapolis, IN) and phosphatase inhibitor (Thermo scientific, Rockford, IL) using a motor homogenizer (TH115, OMNI International, Marietta, GA). Sample lysates were incubated on a rotator at 4°C for 1 hour and the pre-cleared by centrifugation at 15,000 *g* for 15 minutes at 4°C. Samples were resolved by 7 or 10% SDS-PAGE and transferred to nitrocellulose membranes (Bio-Rad, Hercules, CA). Blots were probed with primary antibodies and proteins were visualized by chemiluminescence (Thermo Scientific, Rockford, IL). The AR (N-20), HSP90 and eIF2α antibodies were from Santa Cruz Biotechnology (Santa Cruz, CA), phospho-eIF2α (Ser51) and phospho-JNK antibodies were from Cell Signaling Technology (Danvers, MA), LC3B antibody was from Novus Biologicals (Littleton, CO), GAPDH, BiP and PDI antibodies were from AbCam (Cambridge, MA), 20S proteasome antibody was from Calbiochem (Gibbstown, NJ) and p62 antibody was from American Research Products (Belmont, MA). Western blot quantification was performed using ImageJ.

### Muscle histochemistry and immunofluorescence

Frozen muscle tissue was sectioned at 5 µm with a cryostat and stained with H&E or NADH. For immunofluorescence, 5 µM frozen sections were stained with an antibody against AR and an Alexa Fluor 594 conjugated secondary antibody (Invitrogen). Confocal images were captured with a Zeiss LSM 510 microscope and a water immersion lens (×63).

### Grip strength analysis

The grip strength meter (Columbus Instruments) was positioned horizontally and mice were lowered toward the apparatus. Mice were allowed to grasp the smooth metal triangular pull bar with their fore limbs only, and then were pulled backward in the horizontal plane. The force applied to the bar at the moment the grasp was released was recorded as the peak tension (kg). The test was repeated 5 consecutive times within the same session, and the highest value from the 5 trials was recorded as the grip strength for that animal.

### Statistics

Statistical significance was assessed by two-tailed Student's *t*-test or by ANOVA with the Newman-Keuls multiple comparison test. The distribution of muscle fiber size was analyzed by Mann-Whitney test. All statistics was performed by the Prism 5 (GraphPad Software, San Diego, CA). *P* values less than 0.05 were considered significant.

## Supporting Information

Figure S1 MyoD and myogenin mRNA expression. Relative expression of MyoD and myogenin mRNAs was determined in proximal hind limb muscle of 12 wk mice (*n* = 5–6/genotype) by qPCR. ***p<0.001 by ANOVA, n. s. = not significant.
(TIF)

Figure S2 20S proteasome expression. Western blot shows expression of 20S proteasome subunit in proximal hind limb muscle. Lower panel shows relative quantification of signal intensity. n. s. = not significant.
(TIF)
