# Supplementary figures and images for: Macroautophagy Is Regulated by the UPR–Mediator CHOP and Accentuates the Phenotype of SBMA Mice
Source: PLoS Genet. 2011 Oct 13;7(10):e1002321. doi: 10.1371/journal.pgen.1002321 (PMC3192827; doi:10.1371/journal.pgen.1002321)

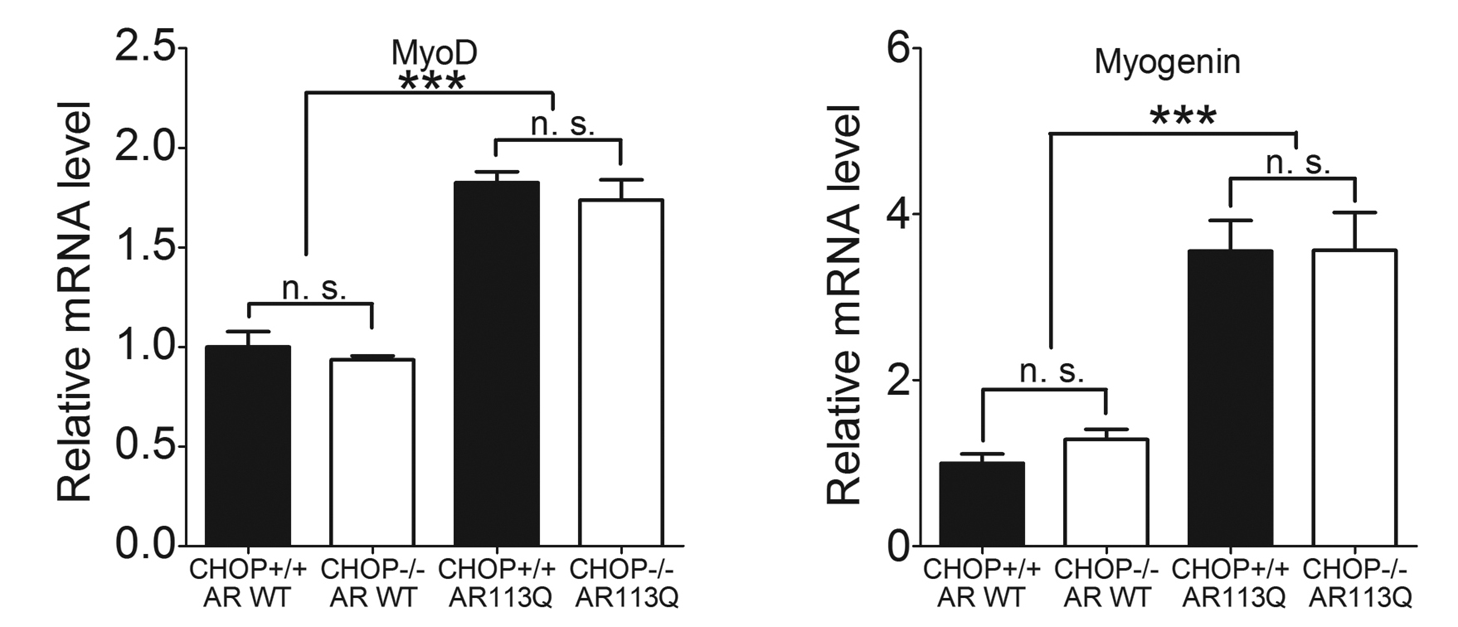

Supplement: Figure S1 — MyoD and myogenin mRNA expression. Relative expression of MyoD and myogenin mRNAs was determined in proximal hind limb muscle of 12 wk mice (n = 5–6/genotype) by qPCR. ***p<0.001 by ANOVA, n. s. = not significant. (TIF) [file pgen.1002321.s001.tif]

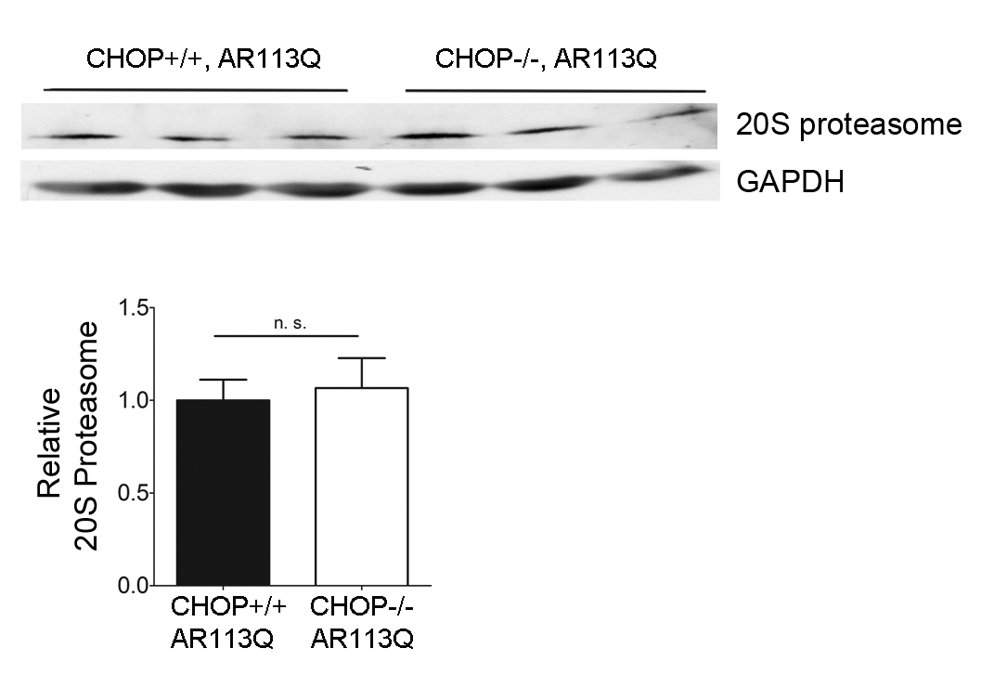

Supplement: Figure S2 — 20S proteasome expression. Western blot shows expression of 20S proteasome subunit in proximal hind limb muscle. Lower panel shows relative quantification of signal intensity. n. s. = not significant. (TIF) [file pgen.1002321.s002.tif]
